# Supplementary material for: Characterization of EIAV env Quasispecies during Long-Term Passage In Vitro: Gradual Loss of Pathogenicity
Source: Viruses. 2019 Apr 24;11(4):380. doi: 10.3390/v11040380 (PMC6520696; doi:10.3390/v11040380)
Supplement: Supplementary file 1 [file viruses-11-00380-s001.pdf]

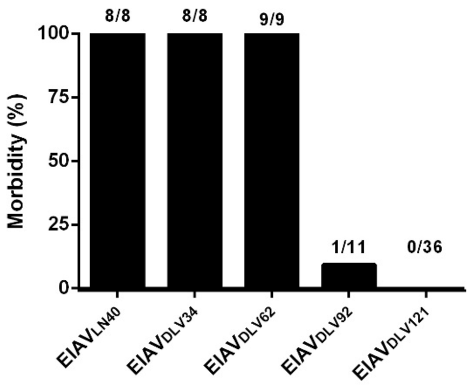

Figure S1. The morbidity of varied EIAV strains. Horses of mixed genders were inoculated with EIAV strains (EIAV$_{LN40}$ 、 EIAV$_{DLV34}$ 、 EIAV$_{DLV62}$ 、 EIAV$_{DLV92}$ and EIAV$_{DLV121}$) by hypodermic injection of 1×10^4 TCID50 of the individual EIAV strains stocks and subsequently all animals were clinically monitored daily (body temperature and platelet). The numbers in parentheses represent the number of horses dead from EIA over the number of horses inoculated (the result from the historical data).

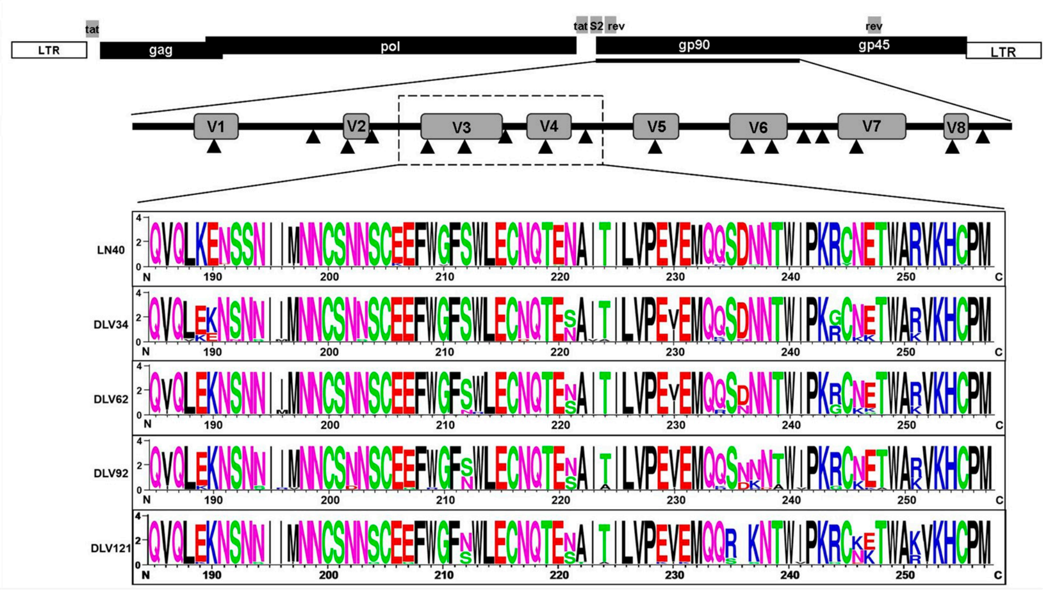

Figure S2. WebLogo presentation of variability in EIAV *env*. The height of each stack shows the level of nucleotide conservation at that position. One letter indicates that the nucleotide at this position is invariant. More letters indicate that that the nucleotide at this position is variable, and all substitutions are noted.

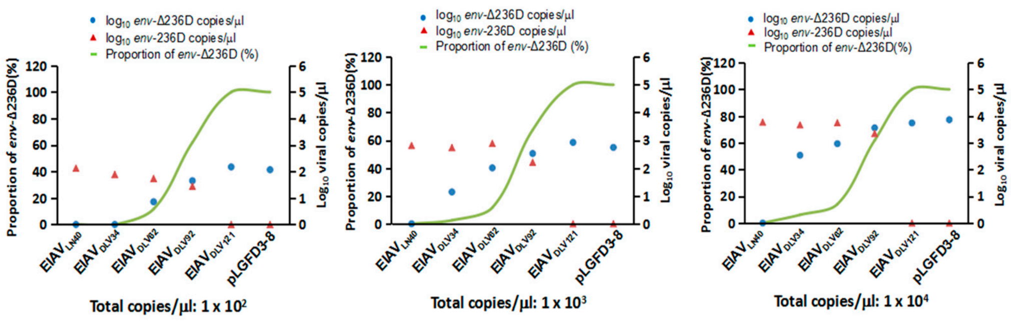

Figure S3. Quantification of the *env*-Δ236D-phenotype and *env*-236D-phenotype sequences located in the V4 region of *env* by double-probe real-time PCR. Three different initial cDNA numbers were simultaneously detected (10^2 copies/μl, 10^3 copies/μl and 10^4 copies/μl). Copies of *env*-Δ236D-phenotype and *env*-236D-phenotype are marked with blue circles and red triangles, respectively. The proportion of *env*-Δ236D-phenotype in the total cDNA copies is shown using a green trend line.
